# Production of Neoagaro-Oligosaccharides With Various Degrees of Polymerization by Using a Truncated Marine Agarase

**DOI:** 10.3389/fmicb.2020.574771

**Published:** 2020-09-24

**Authors:** Wu Qu, Dingquan Wang, Jie Wu, Zhuhua Chan, Wenjie Di, Jianxin Wang, Runying Zeng

**Affiliations:** ^1^Marine Science and Technology College, Zhejiang Ocean University, Zhoushan, China; ^2^Third Institute of Oceanography, Ministry of Natural Resources, Xiamen, China; ^3^Technical Innovation Center for Utilization of Marine Biological Resources, Ministry of Natural Resources, Xiamen, China

**Keywords:** truncated agarase, neoagaro-oligosaccharide, various polymerization degrees, fermentation optimization, recombinant expression

## Abstract

Bioactivities, such as freshness maintenance, whitening, and prebiotics, of marine neoagaro-oligosaccharides (NAOS) with 4–12 degrees of polymerization (DPs) have been proven. However, NAOS produced by most marine β-agarases always possess low DPs (≤6) and limited categories; thus, a strategy that can efficiently produce NAOS especially with various DPs ≥8 must be developed. In this study, 60 amino acid residues with no functional annotation result were removed from the C-terminal of agarase AgaM1, and truncated recombinant AgaM1 (trAgaM1) was found to have the ability to produce NAOS with various DPs (4–12) under certain conditions. The catalytic efficiency and stability of trAgaM1 were obviously lower than the wild type (rAgaM1), which probably endowed trAgaM1 with the ability to produce NAOS with various DPs. The optimum conditions for various NAOS production included mixing 1% agarose (w/v) with 10.26 U/ml trAgaM1 and incubating the mixture at 50°C in deionized water for 100 min. This strategy produced neoagarotetraose (NA4), neoagarohexaose (NA6), neoagarooctaose (NA8), neoagarodecaose (NA10), and neoagarododecaose (NA12) at final concentrations of 0.15, 1.53, 1.53, 3.02, and 3.02 g/L, respectively. The NAOS served as end-products of the reaction. The conditions for trAgaM1 expression in a shake flask and 5 L fermentation tank were optimized, and the yields of trAgaM1 increased by 56- and 842-fold compared with those before optimization, respectively. This study provides numerous substrate sources for production and activity tests of NAOS with high DPs and offers a foundation for large-scale production of NAOS with various DPs at a low cost.

## Introduction

Agarose, the cell-wall component of marine algae, is a polysaccharide that consists of alternating disaccharide units of d-galactose and 3,6-anhydro-l-galactose (Ramos et al., 2016). Hydrolysis products of agarose, namely agaro-oligosaccharides (AOS) and neoagaro-oligosaccharides (NAOS), exhibit potential uses in food, pharmaceutical, and cosmetic industries (Chen et al., 2009; Fu and Kim, 2010; Higashimura et al., 2013, 2016). Acid hydrolysis is a traditional method of degrading agarose into AOS (Xu et al., 2017), and alkalis are used to neutralize the pH value of AOS hydrolysate. However, a large amount of acids and alkalis used during the production process of oligosaccharides can be very harmful to the environment. Agarases from marine microorganisms, which can hydrolyze agarose under mild and environment-friendly conditions, are alternative solutions for acid hydrolysis to produce agarose-derived oligosaccharides (Lee et al., 2012). Agarases are mainly from marine environments and are classified as α- and β-agarases based on cleavage sites. Similar to the acids in hydrolysis, α-agarases cut the α-1,3-glycosidic bond in agarose to produce AOS, and β-agarases cut the β-1,4-glycosidic bond to produce NAOS (Yun et al., 2017). Therefore, enzymatic hydrolysis by β-agarases may be the most feasible method of NAOS production.

Agaro-oligosaccharides (AOS) and NAOS perform many biological activities. For instance, AOS possesses anti-inflammatory, carcinostatic, antioxidant, hepatoprotective, and α-glucosidase inhibitory properties (Chen et al., 2006; Enoki et al., 2010; Higashimura et al., 2013). NAOS exhibits hepatoprotective, freshness maintenance, skin whitening, and cholesterol-lowering activities (Hou et al., 2014; Ji et al., 2017; Jin et al., 2017; Kim et al., 2017). To compare the activities of AOS and NAOS, Xu et al. (2017) studied the antioxidant capacities of agar-derived oligosaccharides and reported that the scavenging ability of NAOS for hydroxyl radicals is greater than that of AOS. Therefore, NAOS might be more effective and valuable than AOS in certain fields.

According to the degree of polymerization (DP), NAOS are categorized as neoagarobiose (NA2), neoagarotetraose (NA4), neoagarohexaose (NA6), neoagarooctaose (NA8), neoagarodecaose (NA10), neoagarododecaose (NA12), and others. Scholars have investigated the bioactivities of NAOS (Hou et al., 2014; Jin et al., 2017). In general, the biological activities of NAOS are correlated with their DPs (Xu et al., 2018). For instance, NA4 and NA6 have high inhibition effects against α-glucosidase, tyrosinase, and melanin biosynthesis (Hong et al., 2017), and the probiotic activities of NA8, NA10, and NA12 are higher than those of NA4 and NA6 (Hu et al., 2006). However, simultaneously outputting these NAOS with various DPs of 4–12 by enzymes is difficult (Hou et al., 2015; Di et al., 2018) due to the problem with most β-agarases, that is, the DPs of NAOS produced by most agarases are usually low (≤6), and the types of NAOS produced by agarases are limited. In a recent study, a β-agarase called AgaXa was isolated from *Catenovulum* sp. X3 and degraded agarose into NA8 and NA10 as main end-products. However, the number of NAOS types is still limited, and almost all β-agarases can only produce 2–3 types of NAOSs (Xie et al., 2013). To solve the problems of simplistic and low DPs of agarase products, researchers mixed AgaXa with another β-agarase that can degrade agarose into NA4 and NA6 to simultaneously produce NAOS with various DPs of 4–12 (Li et al., 2007). However, the cost of fermenting two enzymes is higher than that of using only one enzyme. Thus, an effective method using a single agarase must be developed to produce NAOS with higher and various DPs.

*agaM1* (GenBank accession number MG280837), a β-agarase gene, was isolated from mangrove sediment microbiome (Qu et al., 2018) and characterized through heterologous expression (Di et al., 2018). In the present study, we truncated AgaM1 and found that the truncated recombinant AgaM1 (trAgaM1) could hydrolyze agarose into NAOS with various DPs (NA4, NA6, NA8, NA10, and NA12) as end-products under specific reaction conditions. Moreover, the reaction conditions were optimized for large-scale production of NAOS. In addition, the production of trAgaM1 expressed in *Escherichia coli* cells was optimized in a shake flask and 5 L fermentation tank. Results provide the foundation for future large-scale production of marine NAOS with various DPs.

## Materials and Methods

### Analysis, Cloning, and Expression of Truncated AgaM1 Gene

In our previous study (Di et al., 2018), the agarase gene *agaM1* was cloned and expressed in *E. coli* cells to obtain recombinant AgaM1 (rAgaM1). The conserved domains in the amino acid sequences of AgaM1 were predicted by using BLASTX on the NCBI website,^1^ and a peptide without any annotation result was removed. PCR was performed to truncate the *agaM1* gene by using a forward primer (5'-CAATACGACTGGGATAACATTGCAATTCC-3') and a reverse primer (5'-ATAGATTTCATCCACAAATGGATTCGGATATATAATTAAATCAG-3'). The PCR product was purified and ligated into the pEASY-Blunt E2 expression vector with the T7 promoter (TransGen Biotech Co. Ltd., China). Ultimately, 180 bp of nucleotide sequences were removed from the 3' end of the *agaM1* gene, resulting in the truncation of 60 amino acid residues from the C-terminal of the original rAgaM1. The truncated recombinant protein was called trAgaM1. *E. coli* BL21(DE3) cells harboring the recombinant plasmid were cultured in lysogeny broth (LB) to an OD600 of 0.6 at 37°C (250 rpm). Then, the cells were induced with 0.1 mmol/L isopropyl β-D-thiogalactoside (IPTG) at 17°C for 14 h (250 rpm) in a 250 ml shake flask with an initial liquid volume of 50 ml, which was set as the original condition for trAgaM1 production before optimization. After ultrasonication of the cells, trAgaM1 was purified using a nickel-affinity chromatography column (HisPur Ni-NTA Spin Column, Thermo Fisher Scientific, MA, United States). The unpurified and purified proteins were analyzed *via* 10% sodium dodecyl sulfate-polyacrylamide gel electrophoresis (SDS-PAGE). In detail, the unpurified samples with and without IPTG induction and three identical samples of purified proteins were loaded for SDS-PAGE. After the electrophoresis, two of the samples were stained by Coomassie brilliant blue, and the lane of the third sample was cut off for the activity stain as follows: sodium dodecyl sulfate (SDS) in the gel was removed by rinsing the gel thrice in phosphate buffer saline (PBS; pH 7.4) for approximately 20 min each time after electrophoresis. Subsequently, the gel was placed on the 1% (w/v) agar plate, and the plate was incubated at 30°C for 12 h. The agar plate was stained with iodine solution, and a clean zone could be observed at the position of trAgaM1. Protein concentration was determined using a Pierce BCA Protein Assay Kit (Thermo Fisher Scientific, MA, United States).

### Enzyme Activity Assay

A total of 18.7 μg of the enzyme solution was mixed with 240 μl of Tris-HCl buffer (pH 9) containing 0.2% (w/v) melted agarose. Agarase activity (U) was defined as the amount of enzyme that produced 1 μmol of reducing sugar per minute under the optimum conditions. The content of reducing sugar was determined through 3,5-dinitrosalicylic acid (DNS) method (Miller, 1959). d-galactose was used for standard curve calculation of the reducing sugar content. The mixture was incubated at 50°C for 10 min and was incubated again at 100°C for 10 min with 750 μl of DNS. The amount of reducing sugar was measured by a spectrophotometer at a wavelength of 550 nm (OD550).

### Characterization of trAgaM1

The optimum temperature and pH of trAgaM1 were assayed by incubating the reaction mixture under different temperatures (20–80°C) and pH buffers of citric acid-sodium citrate (0.1 mol/L citric acid, 0.1 mol/L sodium citrate, pH 3–6), PBS (0.2 mol/L Na_2_HPO_4_, 0.3 mol/L NaH_2_PO_4_, pH 7–8), and Tris-HCl (0.1 mol/L Tris base, pH 8–10). Agarase activity under the optimum condition was defined as 100%. The enzyme solution was incubated at 40 and 50°C for 3, 6, 12, 24, 36, and 60 h to determine the thermal stability of trAgaM1. Enzyme activity was measured using the aforementioned method. trAgaM1 activity without any treatment was defined as 100%. The effects of metal ions were assessed by determining trAgaM1 activity with CaCl_2_, NiSO_4_, FeCl_2_, BaCl_2_, ZnSO_4_, KCl, AgNO_3_, MnSO_4_, CdCl_2_, CrCl_2_, SrCl_2_, CuCl_2_, NaCl, and Mg(NO_3_)_2_ at a final concentration of 1 mmol/L. trAgaM1 activity in the absence of any treatment was defined as 100%. All experiments were performed in triplicate.

Agarase activity was assayed at agarose concentrations of 2, 3, 4, 5, 6, 7, and 8 mg/ml under the optimum conditions to determine the kinetic parameters of trAgaM1. *Km* and *Vmax* were calculated using Lineweaver-Burke double reciprocal plot. Thin-layer chromatography (TLC) and high-performance liquid chromatography (HPLC) were used to identify the products of trAgaM1. The standards of NAOS and the products of trAgaM1 were placed into a silica gel 60 TLC plate (Merck, Darmstadt, Germany) and spread using a solvent system containing n-butanol/acetic acid/water (2:1:1; v/v/v). The spots of the products were visualized by spraying 10% (v/v) H_2_SO_4_ and heating them at 100°C for 10 min. For HPLC analysis (Waters Corporation, MA, United States), the mobile phase was 0.005 mol/L H_2_SO_4_. The column temperature was stabilized at 50°C. The flow rate of the mobile phase was 0.6 ml/min. The injection volume was 10 μl, and differential refractive index detector was used for NAOS detection. The HPLC column used for NAOS analysis was Aminex resin-based columns (Bio-Rad, MA, United States).

### Determination of Substrate Utilization Rate of trAgaM1

Substrate utilization rate (SUR) was used as a key index for optimizing the production of NAOS with various DPs. In brief, a reduced dose of trAgaM1 was used to produce NAOS with high and various DPs at 50°C, and Tris-HCl (pH 9) was replaced by deionized water (pH 7.1) to prepare the agarose solution. Then, 200 ml of the reaction solution containing agarose (0.5, 1, 1.5, and 2%; w/v) and enzyme were incubated at 50°C for 3 h to measure the SUR of trAgaM1 under different conditions during optimization. The reaction solution was incubated on ice for 30 min and was centrifuged at 6,000 rpm for 15 min. After centrifugation, the supernatant was discarded, and the remaining agarose gel was resuspended with water and transferred to the original shake flask. The solution in the shake flask was dried in an oven at 80°C. The weight of flask was recorded every 24 h until the value did not change, and the constant weight value was used for the calculation of SUR. The weights of the shake flask before and after the experiment were measured using an electronic analytical balance. SUR was calculated as follows: SUR = (weight of shake flask after the experiment − weight of shake flask before the experiment)/original weight of agarose. The reaction solution containing inactivated trAgaM1 in boiling water bath was subjected to the same procedure and served as the control group. All experiments were performed in triplicate.

### Optimization of trAgaM1 Expression in Shake Flask

The basic medium components for trAgaM1 expression were optimized from the following liquid mediums: LB (10 g of tryptone, 5 g of yeast extract, 10 g of NaCl, and 1 L of H_2_O), super optimal broth (SOB, 20 g of tryptone, 5 g of yeast extract, 0.5 g of NaCl, and 1 L of H_2_O), super optimal broth with catabolite repression (SOC, 20 g of tryptone, 5 g of yeast extract, 0.5 g of NaCl, 2.5 mmol/L KCl, 10 mmol/L MgCl_2_, 20 mmol/L glucose, and 1 L of H_2_O), and 2x YT growth medium (16 g of tryptone, 10 g of yeast extract, 5 g of NaCl, and 1 L of H_2_O). The pH of these mediums was adjusted to 7.4 with NaOH and HCl (approximately 0.1 mol/L). The liquid medium was added with MgCl_2_ (0, 10, 20, 30, and 40 mmol/L), glucose (1%, w/v), lactose (1%, w/v), maltose (1%, w/v), sucrose (1%, w/v), and glycerol (1%, w/v) to optimize trAgaM1 expression. The induction conditions including IPTG concentrations (0, 0.01, 0.1, 1, and 2 mmol/L) and induction temperatures (17, 22, 27, 32, and 37°C) were also optimized. Three natural osmolytes, namely proline, glutamate, and betaine, were added to the broth to a final concentration of 20 mmol/L (with and without 0.5 mol/L NaCl) to reduce the production of inclusion bodies. The feeding strategy was preliminarily studied. Briefly, 50 ml of 1.6% tryptone, 1% yeast extract, 10 mmol/L MgCl_2_, 1% glycerol, and 30 mmol/L proline were fed into the broth after induction for 4 h. The production of trAgaM1 was conducted in a 250 ml shake flask with an initial liquid volume of 50 ml. The strain was first cultured to an OD600 value of 0.6 at 37°C (250 rpm), and the induction time was 16 h (250 rpm). After induction, the cells were broken by ultrasonic wave for assay of agarase activity. The agarase activity (U) per liter of the fermentation broth (total agarase activity, U/L) and the activity (U) produced per gram of the dry cells (specific productivity, U/g) were determined.

### Preliminary Study on Production of trAgaM1 in 5 L Fermentation Tank

The components of the fermentation medium in the 5 L fermentation tank were as follows: 32 g of tryptone, 20 g of yeast extract, 10 g of NaCl, 200 g of glucose, 3.7 g of glycerol, 4.9 g of MgCl_2_, 6.9 g of proline, 0.2 g of ampicillin, and 2 L of H_2_O. The feeding medium contained 600 g of glucose, 10 g of yeast extract, 10 g of MgCl_2_, 3.5 g of proline, 0.1 g of ampicillin, and 1 L of H_2_O. First, 50 ml of bacterial culture with OD600 of 0.8 was inoculated into the fermentation medium and cultured at 37°C with a minute ventilation volume of 10 L and an agitator speed of 500 rpm. The broth pH was adjusted to 7.4 by adding 50% ammonia. The feeding medium was added into the fermentation medium after 6 h of inoculation at 2.5 ml/min. The broth was induced with IPTG at a final concentration of 0.1 mmol/L after 15 h of inoculation. Samples were taken every 3 h to determine total agarase activity and specific productivity.

## Results

### Purification and Characterization of trAgaM1

**Figure 1:**
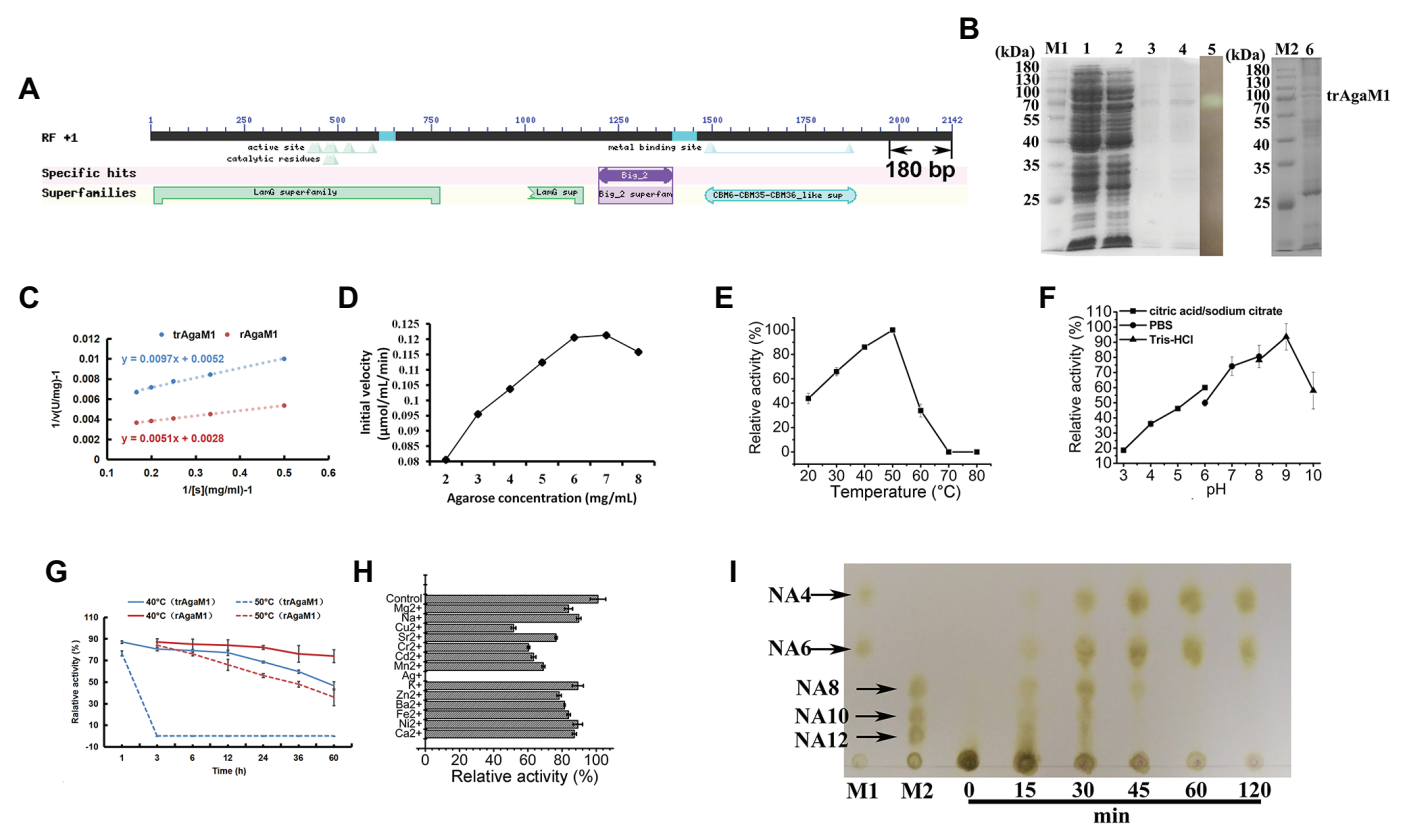
**(A)** Conserved domains annotated in NCBI database and truncated peptides in AgaM1. **(B)** Analysis of truncated recombinant AgaM1 (trAgaM1) by 10% sodium dodecyl sulfate-polyacrylamide gel electrophoresis (SDS-PAGE). Lanes M1 and M2 represent the protein molecular standards; lanes 1 and 2 represent the supernatant of cell disruption liquid without and with isopropy-β-D-thiogalactoside (IPTG) induction, respectively. Lanes 3 and 4 show the purified trAgaM1, and lane 5 is the result of activity stain of trAgaM1 by iodine solution. Samples in lanes 3–5 are identical. Lane 6 shows the proteins in the form of inclusion bodies. **(C)** Determination of kinetic parameters of trAgaM1 by using Lineweaver-Burk plot. The result of trAgaM1 is compared with that of untruncated rAgaM1 from our previous study (Di et al., 2018). **(D)** Initial velocities of trAgaM1 at different agarose concentrations. **(E)** Effect of temperature on trAgaM1 activity. **(F)** Effect of pH on trAgaM1 activity. **(G)** Stability of trAgaM1 against 40 and 50°C. The result of trAgaM1 is compared with that of untruncated rAgaM1 from our previous study (Di et al., 2018). **(H)** Effect of metal ions on trAgaM1 activity. **(I)** Products of trAgaM1 as determined by thin-layer chromatography (TLC) with excess enzyme. Lanes M1 and M2 represent the molecular standard mixtures of neoagaro-oligosaccharides (NAOS) with polymerization degrees of 4–6 and 8–12, respectively.

Our study removed 60 amino acid residues without any annotation result on the NCBI database from the C-terminal of rAgaM1 (Figure 1A). The purified trAgaM1 was analyzed by 10% SDS-PAGE. The result showed that the molecular weight of trAgaM1 was approximately 70 kDa, which was consistent with the theoretical molecular weight of trAgaM1 (71.63 kDa; Figure 1B). The leaked expression was observed because the target protein band was also shown in the control group without IPTG induction, and the inclusion bodies were formed during the expression (Figure 1B). The concentration of the purified trAgaM1 was 80.53 mg/ml. The *Km* and *Vmax* values of trAgaM1 for agarose were 1.87 mg/ml and 192.31 U/mg, respectively (Figure 1C). The initial velocity of trAgaM1 reached a saturation point for agarose at a concentration of 6 mg/ml, and higher substrate concentration (8 mg/ml) showed an inhibition trend for the reaction velocity (Figure 1D). The optimum temperature and pH for trAgaM1 were 50°C and 9, respectively (Figures 1E,F). trAgaM1 maintained 87.1, 80.5, 79.1, 77.2, and 68.6% of the agarase activity after incubation for 1, 3, 6, 12, and 24 h, respectively, at 40°C. This finding indicated the good stability of trAgaM1 at 40°C. However, the agarase activity of trAgaM1 was lost after 3 h of incubation at 50°C (Figure 1G). Heavy metal ions, including Cu^2+^, Sr^2+^, Cr^2+^, Cd^2+^, Mn^2+^, Ag^+^, and Ba^2+^, inhibited the trAgaM1 activity (Figure 1H). When excess enzyme was used, agarose was ultimately hydrolyzed into NA4 and NA6 within 60 min (Figure 1I).

### Optimization of NAOS Production With Various DPs

**Figure 2:**
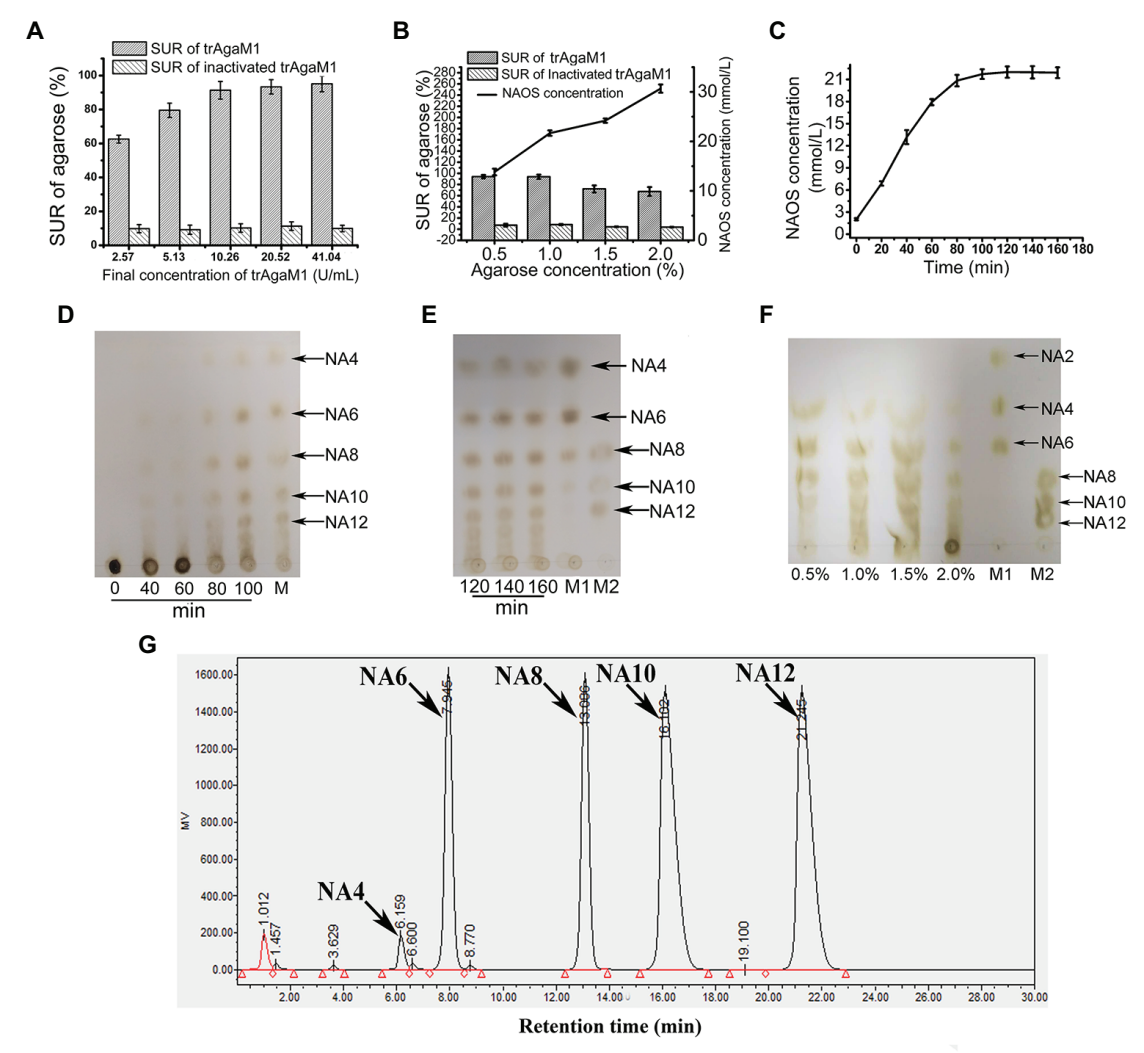
**(A)** Optimization of trAgaM1 dose for the production of NAOS based on substrate utilization rate (SUR). **(B)** Optimization of agarose concentration for the production of NAOS based on SUR and yield of NAOS. **(C)** Optimization of reaction time for NAOS production based on NAOS yield. **(D)** trAgaM1 products from 0 to 100 min under optimized condition. M represents the molecular standard mixtures of NAOS with degrees of polymerization (DPs) of 4–12. **(E)** trAgaM1 products from 120 to 160 min under optimized condition. Lanes M1 and M2 represent the molecular standard mixtures of NAOS with DPs of 4–10 and 8–12, respectively. **(F)** Products of different agarose concentrations degraded by trAgaM1. **(G)** High-performance liquid chromatography (HPLC) result shows the composition and proportion of products degraded by trAgaM1 under optimum conditions. NA4, neoagarotetraose; NA6, neoagarohexaose; NA8, neoagarooctaose; NA10, neoagarodecaose; NA12, neoagarododecaose.

Although excess trAgaM1 can completely degrade agarose into NA4 and NA6, the products of trAgaM1 were maintained at NAOS with DPs of 4–12 (Figure 2D) with a reduced dose of trAgaM1. Therefore, the production of NAOS with various DPs was optimized for large-scale NAOS generation, especially those with DPs higher than six.

Discharge and filtration are needed after the hydrolysis reaction for the further preliminary purification of NAOS in the mass production. However, unhydrolyzed agarose and agar after degradation by trAgaM1 would re-solidify into colloidal solid when the temperature is less than approximately 40°C, thereby blocking the discharge pipes and apertures of the filter membranes. When clogging occurs, reheating is needed to remove the colloid, and the low SUR results in a waste of raw materials and an increase in labor costs. Thus, ensuring the degradation of most of the substrates is important for NAOS production, and SUR is the first factor to be considered. First, the dose of trAgaM1 used for NAOS production was optimized (Figure 2A). The SUR values increased with the trAgaM1 concentration; however, this value did not obviously increase when the final concentration of trAgaM1 was 10.26 U/ml. In consideration of the cost of trAgaM1 production, 10.26 U/ml was chosen as the optimized dose of trAgaM1 for NAOS production. Then, the agarose concentration was optimized. The NAOS production was improved by increasing the agarose concentration. However, the SUR values were 72 and 67.5% at agarose concentrations of 1.5 and 2%, respectively (Figure 2B), indicating that approximately 30% of agarose remained unreacted. The product DPs (4–12) were similar at agarose concentrations of 0.5, 1, and 1.5%; however, the DPs of products remained higher at a concentration of 2% than other agarose concentrations (Figure 2F). At concentrations of 0.5 and 1%, the SUR was high, and a higher amount of NAOS was produced with 1% agarose (Figure 2B). Therefore, 1% agarose was the most suitable for production because of its high NAOS yield and SUR.

Lastly, the reaction time was optimized. In theory, the NAOS yield increases with reaction time. However, the yield becomes stable at a certain level due to substrate reduction and enzyme activity loss. Further increasing the reaction time becomes meaningless. Thus, 200 ml of solution containing 10.26 U/ml (final concentration) trAgaM1 and 1% (w/v) agarose was incubated at 50°C for 3 h. The NAOS yield increased until 100 min (Figure 2C). The SUR of agarose at 100 min was 92.5 ± 2.87%, indicating that almost all of the substrates were degraded. Therefore, 100 min was the optimum reaction time for production.

Samples from the reaction solution under the aforementioned conditions were obtained and subjected to TLC for analyzing the DP of NAOS. NAOS with DPs of 4–12 were produced at 80 min, and the products remained stable from 80 to 160 min (Figures 2D,E). Thus, NAOS with various DPs were produced and served as the end-products of trAgaM1. In summary, the optimum conditions for producing NAOS with various DPs included mixing of 53.35 μg trAgaM1 at a final concentration of 10.26 U/ml with 1% agarose solution and incubating the mixture at 50°C for 100 min. Under the optimized condition, NAOS with DPs of 4–12 were produced with a yield of 9.25 g/L by per gram of trAgaM1. The yield ratio of these NAOS with DPs of 4–12 was 1.6:16.5:16.5:32.7:32.7 based on the peak area of each oligosaccharide (Figure 2G). Therefore, this strategy produced NA4, NA6, NA8, NA10, and NA12 at final concentrations of 0.15, 1.53, 1.53, 3.02, and 3.02 g/L, respectively.

### Production Optimization of trAgaM1 in Shake Flask

**Figure 3:**
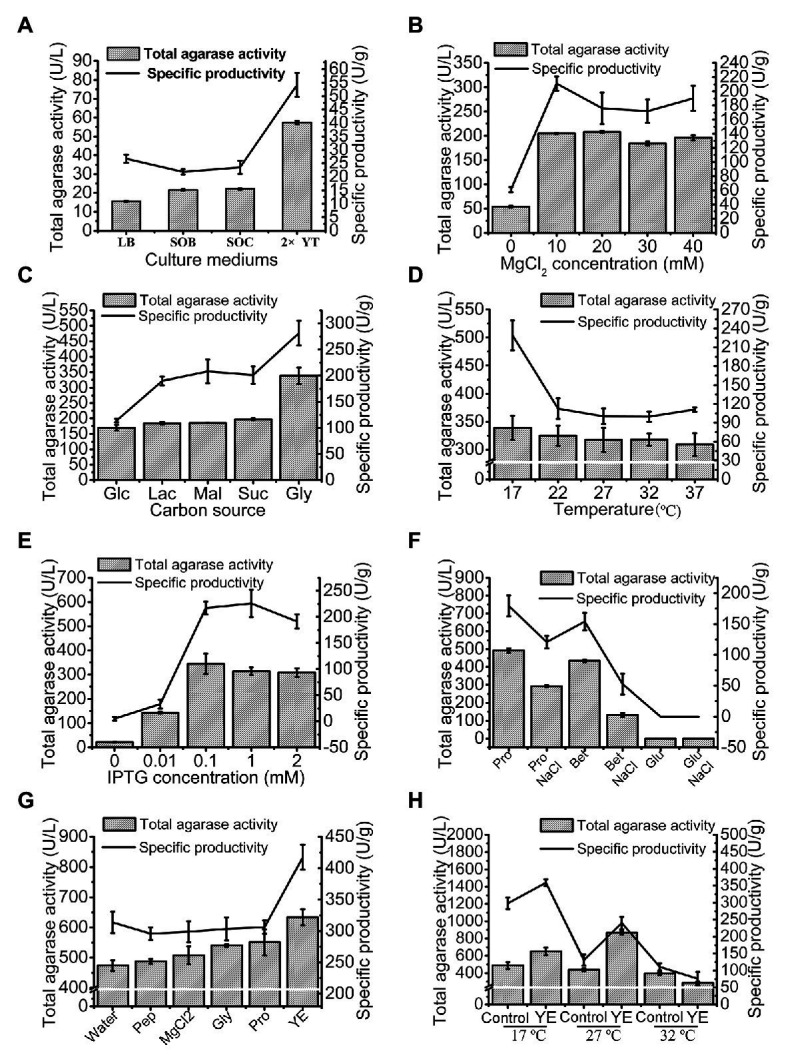
Optimization of trAgaM1 expression in shake flask, including optimization of culture media **(A)**, MgCl_2_ concentrations **(B)**, carbon sources **(C)**, induction temperatures **(D)**, IPTG concentrations **(E)**, additional natural osmolytes **(F)**, and feeding strategy **(G)**. The induction temperatures were re-optimized after the use of 20 mmol/L proline and feed strategy; the feeding groups without the use of proline served as controls **(H)**. Glc, glucose; Lac, lactose; Mal, maltose; Suc, sucrose; Gly, glycerol; Pro, proline; Bet, betaine; Glu, glutamate; Pep, peptone; and YE, yeast extract.

The total agarase activity of 15.51 U/L was produced under the original production conditions. The culture medium was first optimized, and 2x YT was found to be the optimum medium for trAgaM1 expression (Figure 3A). The production of trAgaM1 increased by 4-fold with the addition of MgCl_2_, and no obvious difference in the total activity was detected at different concentrations (10–40 mmol/L). However, MgCl_2_ concentration affected the specific productivity of trAgaM1, and the maximum value was detected at 10 mmol/L (Figure 3B). Therefore, 10 mmol/L MgCl_2_ was supplemented into the culture medium for succeeding experiments. Glycerol (1%) was the optimum carbon source for trAgaM1 expression, leading to a total activity of approximately 1.5-fold greater than that with other carbon sources (Figure 3C). Therefore, 1% glycerol was used as the carbon source for subsequent optimization. The highest total activity and specific productivity were recorded at an induction temperature of 17°C (Figure 3D). IPTG at a concentration of 0.1 mmol/L was considered as the optimum condition for trAgaM1 production (Figure 3E). Accordingly, 0.1 mmol/L IPTG and 17°C were selected as the induction concentration and temperature for subsequent optimization. Furthermore, a leaked expression was observed because 22.08 U/L of total activity was still detected without any IPTG induction (Figure 3E), which is in accordance with the SDS-PAGE result (Figure 1B). Natural osmolytes (with and without 0.5 mol/L NaCl), including proline, glutamate, and betaine, were added into the reaction solution to reduce the production of inclusion bodies. NaCl and glutamate had negative effects on trAgaM1 production. However, betaine and proline without NaCl increased the production of trAgaM1. Furthermore, proline (20 mmol/L) increased the total activity and specific productivity to 492.23 U/L and 179.65 U/g, respectively. Therefore, the optimum medium components for trAgaM1 production included 2x YT medium, 10 mmol/L MgCl_2_, 1% glycerol, and 20 mmol/L proline (Figure 3F).

The feeding strategy was preliminarily studied to improve the trAgaM1 production. Feeding yeast extract increased the total activity and specific productivity to 634.54 U/L and 417.46 U/g (Figure 3G), respectively. For large-scale production of trAgaM1, fed-batch fermentation was performed in a 5 L fermentation tank. However, the optimum induction temperature, which is lower than the normal temperature of water (approximately 25°C), was not reached due to the lack of refrigerating equipment in our fermentation tank. Therefore, the induction temperature was re-optimized. The presence of proline reduced the production of inclusion bodies at high temperatures. Therefore, the amount of trAgaM1 expressed in the supernatant with proline at 27°C was greater than that without proline at 27°C and with proline at 17°C (Figure 3H). Thus, 27°C was the optimum temperature for trAgaM1 production with the optimum medium components.

In summary, the optimum conditions for trAgaM1 production were as follows: induction of *E. coli* cells in the optimum medium (2x YT medium, 10 mmol/L MgCl_2_, 1% glycerol, and 20 mmol/L proline) at 27°C with 0.1 mmol/L IPTG and feeding 50 ml of 1% yeast extract after induction for 4 h. Under these conditions, the total agarase activity and specific productivity of trAgaM1 increased by 56- and 9-fold after optimization, respectively.

### trAgaM1 Production Through Fed-Batch Fermentation

**Figure 4:**
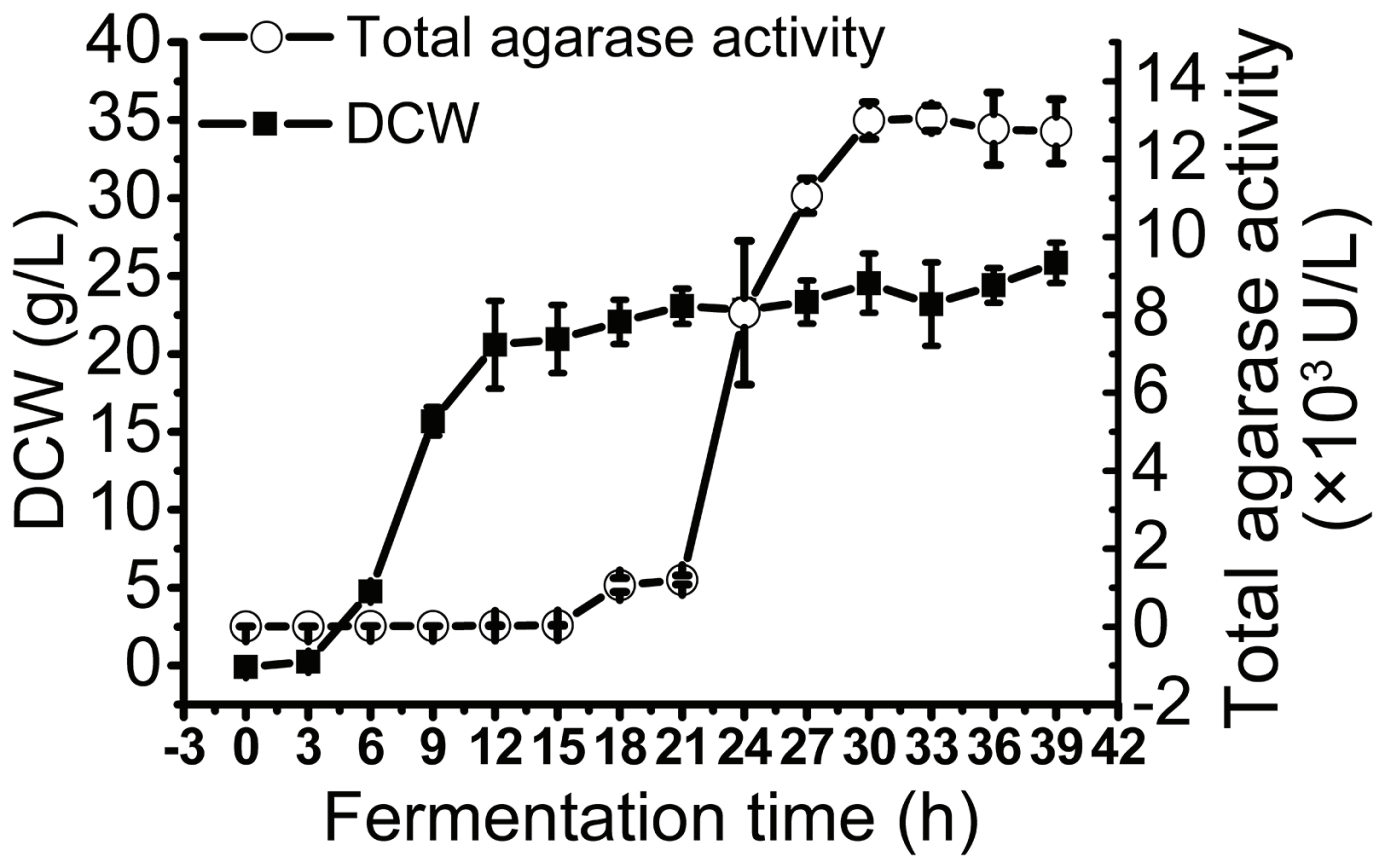
Production of trAgaM1 in 5 L fermentation tank with glucose fed-batch strategy. DCW, dry cell weight.

To obtain large quantities of trAgaM1, we expressed the recombinant protein in a 5 L fermentation tank through a fed-batch strategy. The result showed that the trAgaM1 yield increased through high-density fermentation. The total agarase activity could reach 13,049.44 U/L, which was 842- and 15-fold higher than those obtained in a shake flask under the original and optimum conditions, respectively (Figure 4).

## Discussion

NAOS with higher DPs might harbor greater biological activities (Xu et al., 2018). However, the bioactivities of NAOS with DPs greater than six have been hardly produced because NAOS with higher DPs are difficult to produce by using available agarases (Hou et al., 2015; Di et al., 2018). The production of NAOS with DPs of four and six was also important due to their biological activities (Jin et al., 2017). Therefore, a strategy for NAOS production with various DPs (e.g., 4–12) is crucial for their future biotechnological applications. In this study, a method to produce NAOS with DPs in the range of 4–12 was established and optimized, which enables the mass production of NAOS with various DPs by using a single enzyme.

Former studies showed that the truncation of amino acids from the C-terminal of agarases could change the enzymatic properties, for instance, the improvement of agarase activity (Ma et al., 2019). Therefore, our work aimed to obtain some special properties by the truncation of the original rAgaM1, and the results showed that NAOS with various DPs were produced by this truncated recombinant agarase, trAgaM1. Compared with the untruncated rAgaM1, two properties of trAgaM1 were varied (Di et al., 2018). First, the *Vmax* (192.31 U/mg) after truncation is 1.86-fold lower before truncation (357.14 U/mg; Figure 1B), suggesting the decreased catalytic efficiency of breaking the β-1,4-glycosidic bond. Second, the thermal stability at 50°C after truncation is weaker than the original agarase. The original agarase rAgaM1 maintained more than 65% of its activity after 10 h of pre-incubation at 50°C (Di et al., 2018). However, the agarase activity of trAgaM1 was completely lost after 3 h of pre-incubation at 50°C (Figure 1C). The effects of sequence deletion on the agarase characteristics are different. A total of 140 amino acid peptides with unknown function were truncated from the C-terminal of AgaG4, causing a 35-fold increase of agarase activity (Liu, 2015); however, the removal of 60 amino acids from AgaM1 in this study decreased the activity and stability of this agarase. Conversely, the truncated AgaO lost all the agarase activity (Han et al., 2016). Without the information of protein structures, these differences cannot be scientifically and detailly explained. However, we speculated that these amino acids might strongly associate with the enzymatic activity and stability.

The optimum pH of trAgaM1 was 9; however, Tris-HCl buffer (pH 9) was replaced by deionized water (pH 7.1) for the production of various NAOS to avoid environmental pollution by alkaline solutions. We speculated that the non-optimum pH, along with the changes of activity and stability, probably provided the ability of trAgaM1 to produce NAOS with various DPs as end-products under certain conditions: although excess trAgaM1 can still degrade agarose into NA4 and NA6 (Figure 1G), some NAOS with higher DPs cannot be further degraded due to the non-optimum pH, weak activity of trAgaM1, and the activity lost throughout the reaction time with reduced amount of enzyme (10.26 U/ml), resulting in the production of NAOS with various DPs. In the previous study, the mutation of three amino acids endows agarase AgaD with the ability to produce NAOS with various DPs (David et al., 2018). The agarase activity of AgaD reduces 50-fold after the mutation (David et al., 2018), which is similar to the result in our study. Further comparison with other works is ineffective due to the scarce related studies. However, we infer that decreasing rather than increasing the activity is propitious to produce NAOS with high and various DPs. Therefore, NAOS with more diverse and higher DPs would be produced by the reformed β-agarase through truncation and/or other forms of amino-acid sequence modification in future works. The present study provides direction toward future NAOS production through enzyme modification.

The conditions for producing NAOS with various DPs were optimized in this study, which successfully resulted in NAOS with the highest SUR and yields. NAOS yield is always considered, whereas SUR is often neglected during production. SUR is greatly important for production because a low SUR results in the waste of substrate and labor cost and the loss of equipment. The SUR value of 92.5% can be reached after optimization. In addition, NAOS with various DPs are the intermediate products of most β-agarases and can be produced by many β-agarases theoretically if the reaction time is precisely controlled. Unfortunately, fully controlling the reaction time in the complex and extensive industrial production is unrealistic. In this study, NAOS with various DPs serve as the end-products under optimized conditions (Figure 2D), and these stable products make the reaction more flexible and easily controlled. In summary, the high SUR and yield of NAOS and the stable final products demonstrate that our method is a promising strategy for large-scale NAOS production.

Furthermore, the expression level of trAgaM1 was optimized to provide sufficient enzymes for NAOS production. During *E. coli* expression, the formation of inclusion bodies usually causes the low productivity of recombinant proteins (Le et al., 2011; Jhamb and Sahoo, 2012). Lowering the induction temperature is the most common method to reduce the production of inclusion bodies. However, refrigeration would consume additional energy during fermentation. Supplementing natural osmolytes, the so-called chemical chaperones, into the fermentation medium is an alternative method to decrease the formation of inclusion bodies (Diamant et al., 2010). Viscous osmolytes can significantly increase the stability of thermolabile proteins and reduce the rate of protein-folding, thereby improving the yields of the natively folded recombinant proteins (Diamant et al., 2001, 2010; de Marco et al., 2005). For the trAgaM1 fermentation, the total agarase activity with proline and betaine was increased by 1.4- and 1.2-fold compared with the treatment without osmolytes (Figures 3E,F). Additional NaCl can induce the osmolyte absorption into *E. coli* cells, which indirectly improves the yields of soluble recombinant proteins. However, trAgaM1 production with osmolytes decreased with additional NaCl regardless of the use of proline and betaine (Figure 3F). This finding was due to the salt concentration in 2x YT (0.5%), which was probably sufficient for osmolyte absorption, and the recombinant protein expression was inhibited by the additional NaCl (Duan et al., 2013). Importantly, proline increased the yield of trAgaM1 at high temperature, which then, increased the optimum induction temperature to 27°C (Figure 3H). This value is close to the room temperature, resulting in significant energy savings during the mass production because that the extra refrigeration and excessive heating were not incurred. In addition, natural osmolytes may also increase the yield of other recombinant proteins.

In conclusion, a strategy that can produce NAOS with various DPs was established and optimized in this study. Through this strategy, NAOS with DPs in the range of 4–12 as end-products can be produced using a single agarase trAgaM1 with high SUR and yield, which enables mass production. Moreover, the production of trAgaM1 was optimized, resulting in a total increase of 842-fold. The potential of natural osmolytes was also discussed based on our results. This study provides many substrate sources for the production and activity tests of NAOS with higher DPs and lays a foundation for the large-scale production of trAgaM1 and NAOS with various DPs at a low cost.

## Data Availability Statement

The datasets presented in this study can be found in online repositories. The names of the repository/repositories and accession number(s) can be found below: https://www.ncbi.nlm.nih.gov/genbank/, MG280837.

## Author Contributions

WQ and RZ designed this study. WQ, DW, and WD performed the experiments. WQ, JW, and DW analyzed the sequencing data. WQ, RZ, and DW wrote the paper. JW revised the paper. All authors contributed to the article and approved the submitted version.

### Conflict of Interest

The authors declare that the research was conducted in the absence of any commercial or financial relationships that could be construed as a potential conflict of interest.

## Footnotes

^1^https://blast.ncbi.nlm.nih.gov/
